# Dietary fat intake and age modulate the composition of the gut microbiota and colonic inflammation in C57BL/6J mice

**DOI:** 10.1186/s12866-019-1557-9

**Published:** 2019-08-20

**Authors:** Su Jeong Kim, Sung-Eun Kim, A-Reum Kim, Saemyi Kang, Mi-Young Park, Mi-Kyung Sung

**Affiliations:** 1 Department of Food and Nutrition, Sookmyung Women’s University, 100, Cheongpa-ro 47-gil, Yongsan-gu, Seoul, 04310 Republic of Korea; 2 Department of Food and Nutrition Education, Graduate School of Education, Soonchunhyang University, Asan, Chungnam 31538 Republic of Korea

**Keywords:** Age, High-fat diet, Gut microbiota, Colonic inflammation

## Abstract

**Background:**

More than half of the adult population worldwide is overweight or obese, while excess adiposity has been linked to chronic low-grade inflammation, contributing to the development of chronic diseases. Recent studies have showed that diet-induced alterations to the gut microbiota composition play a pivotal role in the development of obesity. However, the cause-effect relationship between obesity and gut microbiota composition is not yet fully understood. In this study, we investigated the short-term responses of gut microbiota composition to diets with different fat contents and their associations with inflammatory biomarkers.

**Results:**

Sixty male C57BL/6 J mice were fed a normal diet (ND; 15% fat) or a high-fat diet (HFD; 45% fat) for 10 or 20 weeks. The relative proportion of the phylum Actinobacteria was elevated by the HFD and was positively associated with body weight and proinflammatory cytokines including TNF-α, IL-1β, and IL-6. The proportion of the phylum Firmicutes increased with aging and was also positively correlated with proinflammatory cytokines. The proportions of Actinobacteria and Firmicutes were inversely associated with tight junction proteins claudin-1 and E-cadherin, respectively. The proportions of the class Clostridia and the family Ruminococcaceae within the phylum Firmicutes were affected by both diet and age. In addition, the proportions of the phylum Bacteroidetes, the family Bacteroidaceae, and the genus *Bacteroides* decreased with aging and were inversely correlated with colonic proinflammatory cytokines representing a positive association with tight junction proteins.

**Conclusions:**

Host age and dietary fat intake are important elements that induce proportional changes in gut microbiota, and these changes are also associated with systemic inflammation. This study provides evidence that diet affects the gut microbiota composition within a short period of time.

## Background

Increased intake of energy-dense foods and sedentary lifestyles have contributed to a sharp increase in the obese population. According to the World Health Organization (WHO), more than half of the adult population is overweight or obese, and excess adiposity is linked to chronic low-grade inflammation, contributing to the development of chronic diseases such as diabetes, nonalcoholic fatty liver, cardiovascular diseases, and certain types of cancer [1, 2]. Interestingly, a growing body of evidence suggests that the composition of bacteria residing in the gastrointestinal tract is related to metabolic disturbances [3].

The gut microbiota colonizes the mucosal layer of different regions of the human gut, with significant interactions taking place between the microbiota and the host [4]. Among the various pathogenic conditions in which the gut microbiota plays a role, obesity is one of the most frequently reported [5–9]. Many studies have suggested that obesity is related to a decrease in the ratio of Bacteroidetes to Firmicutes. However, other studies have shown that there was no difference in this ratio between obese and non-obese subjects [10–12]. In another study of obese subjects, the proportion of Bacteroidetes was found to be decreased, whereas the proportion of Actinobacteria was increased [11]. Therefore, the association between obesity and specific microbial phyla largely remains controversial.

The gut microbiome has been suggested to be a causative factor in the development of obesity in a number of rodent model studies. Germ-free mice colonized with the gut microbiota from conventionally raised mice showed higher body fat contents and increased insulin resistance [13]. A follow-up study indicated that the gut microbiota suppressed the intestinal expression of a lipoprotein lipase (LPL) inhibitor, fasting-induced adipose factor (Fiaf), and adenosine monophosphate-activated protein kinase (AMPK)-driven fatty acid oxidation in the liver and skeletal muscle, thus promoting the accumulation of adipocyte triglycerides [14]. In addition, the gut microbiota ferment dietary fiber to produce short chain fatty acids, which provide microbiota-generated calories [15]. Although rodent model studies suggest that alterations to the gut microbiome causally regulated obesity development, it has been well established that environmental factors, particularly diet, can be powerful modulators of gut microbiome composition. Therefore, the complexity of the cause-effect association between the gut microbiome and the development of obesity is far greater than one might expect.

A recent review indicated that a high-fat diet (HFD) before the onset of obesity induces intestinal dysbiosis, contributing to low grade inflammation, decreased expression of anti-microbial peptides, mucus layer depletion, and decreased expression of gap junction proteins, which allows barrier disruption and the passage of bacterial components, activating secondary immune responses and creating metabolic complications [16]. Therefore, HFD-induced metabolic complications might be mediated by gut dysbiosis and associated inflammatory responses. The objective of this study was to investigate the short-term response of the gut microbiome profile to a HFD and to identify specific microbes associated with age, dietary fat content, and pro-inflammatory biomarkers.

## Results

### Body weights of animals

**Fig. 1:**
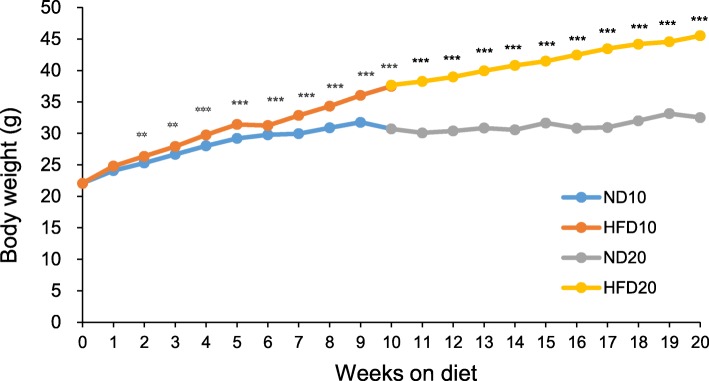
Effect of dietary fat intake on body weight. Data are means ± SEM. The statistical significance of differences was evaluated by Student’s t-test (** *P* < 0.01, *** *P* < 0.001). ND10, normal fat diet for 10 weeks (*n* = 15); HFD10, high-fat diet for 10 weeks (*n* = 15); ND20, normal fat diet for 20 weeks (*n* = 15); and HFD20, high-fat diet for 20 weeks (*n* = 16)

Figure 1 shows the average body weights of the experimental animals in each diet group. The body weights of mice fed the HFD were significantly higher than the weights of mice fed the normal diet (ND) after only 2 weeks (*P* < 0.01), and this significant difference was maintained over the experimental period. At week 20, the body weights of the animals in the HFD20 group were significantly higher than the weights of animals in the ND20 group (*P* < 0.01).

### Colonic mRNA expressions of proinflammatory cytokines and tight junction proteins

To test for a proinflammatory shift and disruption to intestinal barrier function, we analyzed the mRNA expression of several proinflammatory cytokines (TNF-α, IL-1β, and IL-6) and the mRNA and protein expressions of tight junction markers (claudin-1, E-cadherin, occludin, and ZO-1).

**Fig. 2:**
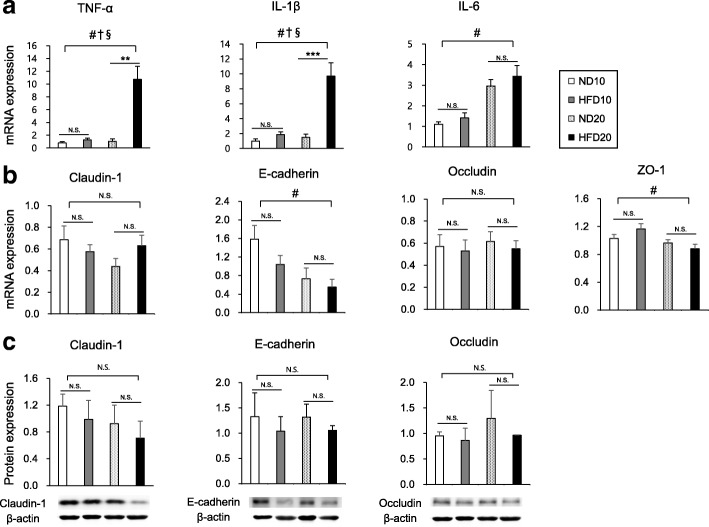
Effect of dietary fat intake on colonic expressions of proinflammatory cytokines (**a**) and tight junction markers (**b** and **c**). Data are means ± SEM. Data were analyzed by Student’s t-test (** *P* < 0.01, *** *P* < 0.001) and two-way ANOVA (#, *P* < 0.05 for age effect; †, *P* < 0.05 for diet effect; §, *P* < 0.05 for the interaction between age and diet). ND10, normal fat diet for 10 weeks (*n* = 15 for **a**, **b** and *n* = 6 for **c**); HFD10, high-fat diet for 10 weeks (*n* = 15 for **a**, **b** and *n* = 6 for **c**); ND20, normal fat diet for 20 weeks (*n* = 15 for **a**, **b** and *n* = 6 for **c**); and HFD20, high-fat diet for 20 weeks (*n* = 16 for **a**, **b** and *n* = 5 for **c**)

The mRNA expression of proinflammatory cytokines including TNF- α, IL-1β, and IL-6 were increased as age increases, whereas those of E-cadherin and ZO-1 were decreased with age (Fig. 2a and b, *P* < 0.05). HFD also significantly increased the mRNA expression of TNF- α and IL-1β (Fig. 2a, *P* < 0.05) and there were significant interactions between age and diet in the expressions of both TNF-α (F_(1,14)_ = 16.84, *P* = 0.0003) and IL-1β (F_(1,14)_ = 4.97, *P* = 0.0332). There were no significant differences in the mRNA and protein expressions of tight junction markers between the ND and HFD groups at both week 10 and week 20, although protein expressions showed tendencies to decrease in HFD groups (Fig. 2b and c).

### Microbial diversity

**Fig. 3:**
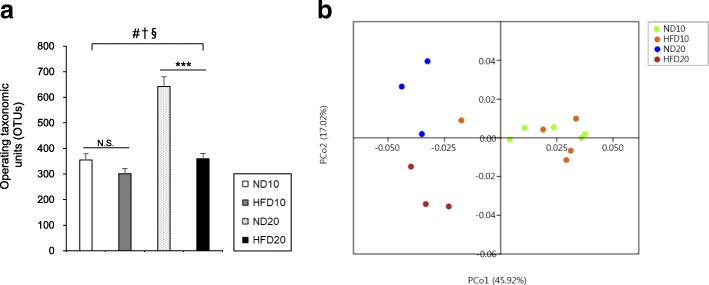
Effect of dietary fat intake on gut microbiota diversity. **a** Operating taxonomic units and **b** Principal coordinate analysis. Data are means ± SEM. Data were analyzed by Student’s t-test (*** *P* < 0.001) and two-way ANOVA (#, *P* < 0.05 for age effect; †, *P* < 0.05 for diet effect; §, *P* < 0.05 for the interaction between age and diet). ND10, normal fat diet for 10 weeks (*n* = 5); HFD10, high-fat diet for 10 weeks (*n* = 5); ND20, normal fat diet for 20 weeks (*n* = 3); and HFD20, high-fat diet for 20 weeks (*n* = 3)

A diversity index is a quantitative measure that reflects how many different species are present in a group. In a phylogenic study, operating taxonomic units (OTUs) are the operational definition of a species or group of species [17], and is a commonly used unit of microbial diversity. OTU richness was higher in ND20 group compared to the HFD20 group and it was influenced by age, diet, and the interaction between diet and age (Fig. 3a, *P* < 0.05). One-way analysis of similarities (ANOSIM) test based on the UniFrac distance matrix showed strong (global *R* = 0.690) and significant (*P* < 0.001) differences in community structure among the sample groups; in the pairwise post hoc test, large and significant differences between ND10 and ND20, ND10 and HFD20, HFD10 and ND20, and HFD10 and HFD20 were reported. Difference in community structure between ND20 and HFD20 was large (*R* = 0.704) but a little significant (*P* = 0.099) (Table 1). These data indicated that age is an important variable for inducing changes in the gut microbiota composition. A principal coordinate analysis (PCoA) plot showed the discrimination between the ND20 and HFD20 groups, with most HFD samples being positioned in the lower part of the plot, suggesting that age and dietary fat content are significant variables. Meanwhile, there was overlap between the ND10 and HFD10 groups (Fig. 3b).

**Table 1:** Analysis of similarities (ANOSIM) representing differences in microbial community structure among groups

	ND10	HFD10	ND20
ND10			
HFD10	0.06		
ND20	1.00**	0.85**	
HFD20	1.00**	0.93**	0.70*

Significance level was marked as *(a little significant, *P* < 0.1) and **(significant, *P* < 0.05). The one-way ANOSIM was performed on UniFrac distance matrix of four groups with 10,000 permutations

### The effects of diet and age on microbial composition

**Fig. 4:**
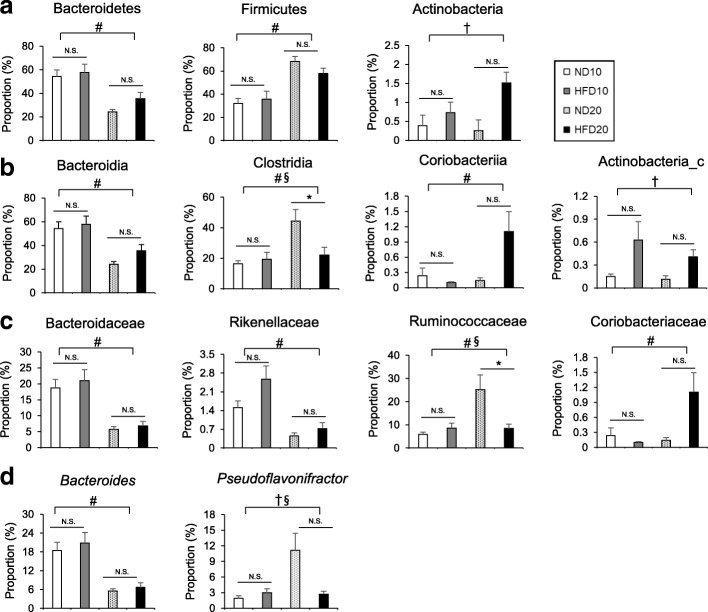
Effects of diet and age on the microbial composition at the phylum (**a**), class (**b**), family (**c**), and genus (**d**) levels. Data are means ± SEM. Data were analyzed by Student’s t-test (*P < 0.05) and two-way ANOVA (#, *P* < 0.05 for age effect; †, *P* < 0.05 for diet effect; §, *P* < 0.05 for the interaction between age and diet). ND10, normal fat diet for 10 weeks (*n* = 5); HFD10, high-fat diet for 10 weeks (*n* = 5); ND20, normal fat diet for 20 weeks (*n* = 3); and HFD20, high-fat diet for 20 weeks (*n* = 3)

To determine the effects of diet, age, and the interaction between diet and age on microbial composition, four groups (the ND10, HFD10, ND20, and HFD20 groups) were analyzed by two-way ANOVA (Fig. 4). Diet significantly influenced the proportions of the phylum Actinobacteria (F_(1,14)_ = 6.12, *P* = 0.0268) and the class Actinobacteria_c (F_(1,14)_ = 6.49, *P* = 0.0232). In the phylum Actinobacteria, age increased the proportions of both the class Coriobacteriia (F_(1,14)_ = 1.47, *P* = 0.0304) and the family Coriobacteriaceae (F_(1,14)_ = 5.80, *P* = 0.0304) (Fig. 4a-c). Within the phylum Bacteroidetes, the class Bacteroidia, the family Bacteroidaceae, the family Rikenellaceae, and the genus *Bacteroides* were significantly affected by age. Age significantly decreased the percentages of Bacteroidetes (F_(1,14)_ = 17.62, *P* = 0.0009), Bacteroidia (F_(1,14)_ = 17.61, *P* = 0.0009), Bacteroidaceae (F_(1,14)_ = 26.46, *P* = 0.0001), Rikenellaceae (F_(1,14)_ = 17.25, *P* = 0.0010), and *Bacteroides* (F_(1,14)_ = 26.95, *P* = 0.0001) in mice (Fig. 4). Meanwhile, age significantly elevated the proportions of Firmicutes (F_(1,14)_ = 26.62, *P* = 0.0001), Clostridia (F_(1,14)_ = 7.19, *P* = 0.0179), and Ruminococcaceae (F_(1,14)_ = 8.29, *P* = 0.0121). The proportion of *Pseudoflavonifractor* was altered by diet (F_(1,14)_ = 8.34, *P* = 0.0119). There was a significant interaction between diet and age within the class Clostridia (F_(1,14)_ = 8.04, *P* = 0.0132), the family Ruminococcaceae (F_(1,14)_ = 14.88, *P* = 0.0017), and the genus *Pseudoflavonifractor* (F_(1,14)_ = 17.20, *P* = 0.0010) (Fig. 4). Overall, microbial composition was generally affected by age rather than diet, while the proportion of Clostridia and Ruminococcaceae was significantly lower in the HFD20 group compared with the ND20 group (Fig. 4b-c). Also, significant interactions between age and diet should be noted to evaluate the effects of these two variables on microbial composition.

### Correlations of gut microbiota with body weight and colonic biomarkers

**Fig. 5:**
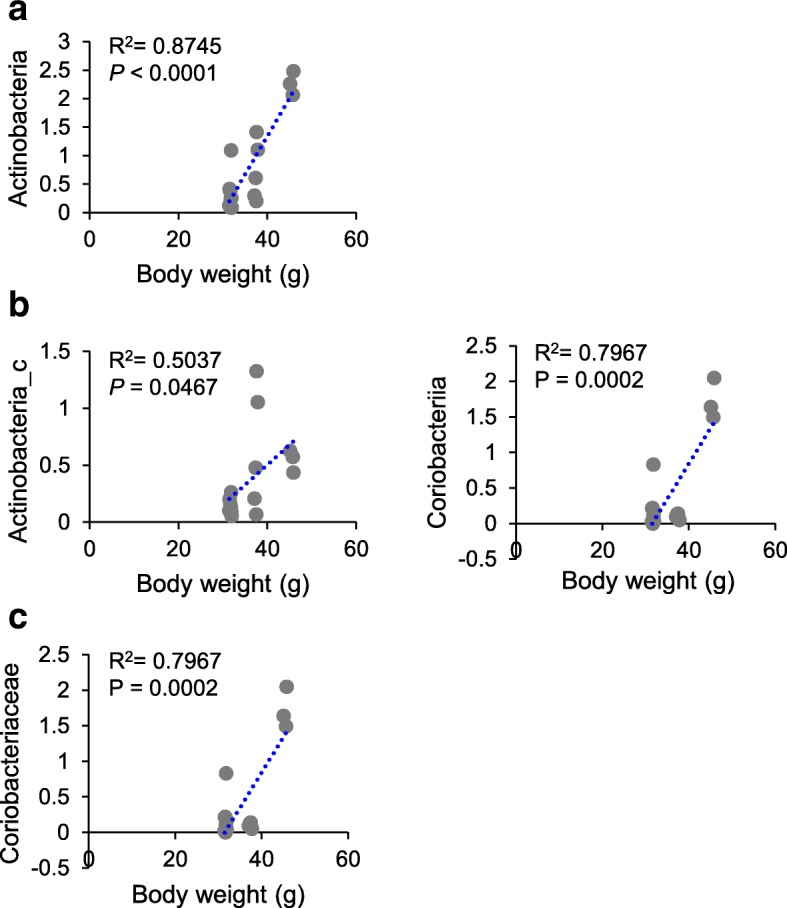
Correlations between relative abundances of microbial taxa with body weight at the phylum (**a**), class (**b**), and family (**c**) levels. Statistical analyses were performed by Pearson’s correlation coefficient. Y-axis, proportion (%)

**Fig. 6:**
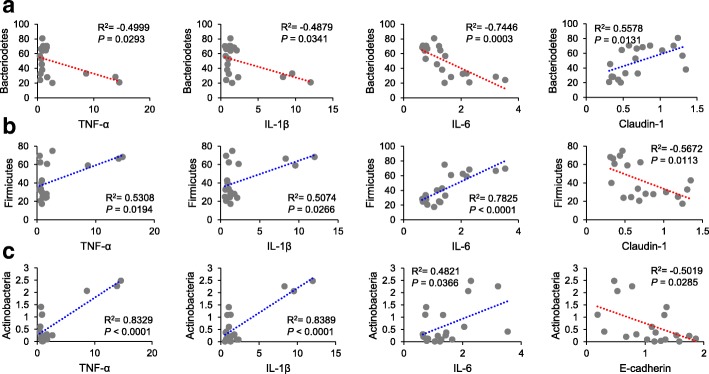
Correlations between relative abundances of the phylum Bacteroidetes (**a**), Firmicutes (**b**), and Actinobacteria (**c**) with proinflammatory cytokines and tight junction proteins at the phylum level. Statistical analyses were performed by Pearson’s correlation coefficient. X-axis, relative expression level; Y-axis, proportion (%)

**Fig. 7:**
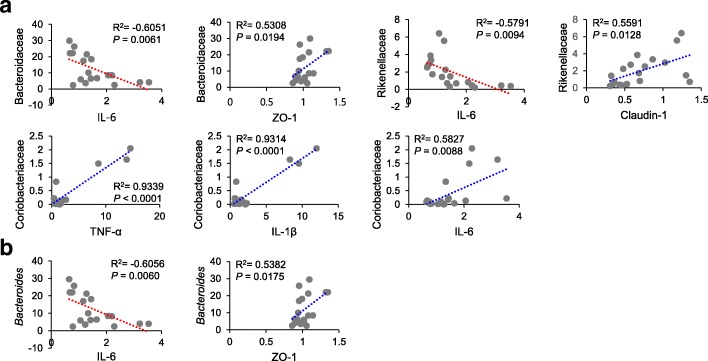
Correlations between relative abundances of microbial taxa with proinflammatory cytokines and tight junction proteins at the family (**a**) and genus (**b**) levels. Statistical analyses were performed by Pearson’s correlation coefficient. X-axis, relative expression level; Y-axis, proportion (%)

To reveal correlations between gut microbiota composition, body weight, and colonic expressions of biomarkers, we examined correlations between relative abundances of bacterial groups with body weight and colonic expressions of proinflammatory cytokines and tight junction proteins. A positive correlation was found between body weight and the relative abundances of the phylum Actinobacteria, the classes Actinobacteria_c and Coriobacteriia*,* and the family Coriobacteriaceae (Actinobacteria, *R*^*2*^ = 0.8745, *P <* 0.0001; Actinobacteria_c, *R*^*2*^ = 0.5037, *P* = 0.0467; Coriobacteriia, *R*^*2*^ = 0.7967, *P* = 0.0002; Coriobacteriaceae, *R*^*2*^ = 0.7967, *P* = 0.0002) (Fig. 5). Furthermore, the proportion of the phylum Bacteroidetes was negatively associated with proinflammatory cytokines (TNF-α, *R*^*2*^ = − 0.4999, *P* = 0.0293; IL-1β, *R*^*2*^ = − 0.4879, *P* = 0.0341; IL-6, *R*^*2*^ = − 0.7446, *P* = 0.0003) and positively correlated with claudin-1 (*R*^*2*^ = 0.5578, *P* = 0.0131) (Fig. 6a). The proportions of the family Bacteroidaceae and the genus *Bacteroides* showed a negative relationship with IL-6 (Bacteroidaceae, *R*^*2*^ = − 0.6051, *P* = 0.0061; *Bacteroides*, *R*^*2*^ = − 0.6056, *P* = 0.0060) and a positive relationship with ZO-1 (Bacteroidaceae, *R*^*2*^ = 0.5308, *P* = 0.0194; *Bacteroides*, *R*^*2*^ = 0.5382, *P* = 0.0175) (Fig. 7a and b). In addition, the proportion of the family Rikenellaceae was inversely associated with IL-6 (*R*^*2*^ = − 0.5791, *P* = 0.0094), while it was positively correlated with claudin-1 (*R*^*2*^ = 0.5591, *P* = 0.0128) (Fig. 7a).

In contrast, the proportion of Firmicutes showed positive relationships with proinflammatory cytokines (TNF-α, *R*^*2*^ = 0.5308, *P* = 0.0194; IL-1β, *R*^*2*^ = 0.5074, *P* = 0.0266; IL-6, *R*^*2*^ = 0.7825, *P* < 0.0001) and a negative relationship with claudin-1 (*R*^*2*^ = − 0.5672, *P* = 0.0113) (Fig. 6b). Similarly, the proportion of Actinobacteria was positively related with proinflammatory cytokines (TNF-α, *R*^*2*^ = 0.8329, *P* < 0.0001; IL-1β, *R*^*2*^ = 0.8389, *P* < 0.0001; IL-6, *R*^*2*^ = 0.4821, *P* = 0.0366) and negatively associated with E-cadherin (*R*^*2*^ = − 0.5019, *P* = 0.0285) (Fig. 6c). In the phylum Actinobacteria, a positive correlation was also found between the family Coriobacteriaceae and proinflammatory cytokines (TNF-α, *R*^*2*^ = 0.9339, *P* < 0.0001; IL-1β, *R*^*2*^ = 0.9314, *P* < 0.0001; IL-6, *R*^*2*^ = 0.5827, *P* = 0.0088) (Fig. 7a).

## Discussion

Numerous studies have suggested that the composition of the gut microbiota differs between obese and normal weight individuals [18–20]. However, the cause-effect relationship between obesity and gut microbiota composition is not yet fully understood. This study investigated the short-term responses of the gut microbiota composition to diets with different fat contents. Experimental animals were fed either a ND or HFD for 20 weeks and the microbial composition was evaluated at 10 and 20 weeks. In agreement with previous studies, body weight and the expression of colonic cytokines increased with higher dietary fat content. The diversity of the gut microbiota was significantly influenced by both age and diet, and two variable showed significant interactions.

At the phylum level, the proportion of Actinobacteria was significantly associated with dietary fat content, while the proportions of Firmicutes and Bacteroidetes were strongly associated with age. In the present study, a HFD significantly elevated the proportions of the phylum Actinobacteria and the class Actinobacteria_c in a positive association with body weight, which have also been shown to be increased in obese subjects and patients with type 2 diabetes [21, 22]. A growing body of evidence suggests that a HFD increases gut permeability and endotoxemia, resulting in low-grade inflammation and impairment of the gut barrier [23–26]. Given that bacteria in the phylum Actinobacteria are known as mucin-degrading bacteria, abundant Actinobacteria might be associated with gut barrier impairment induced by a HFD [27]. Indeed, we observed that Actinobacteria was inversely related with tight junction proteins such as E-cadherin and positively associated with proinflammatory cytokines. Therefore, the HFD-mediated increase in Actinobacteria and Actinobacteria_c may play a role in the HFD-induced impairment of the intestinal barrier, leading to colonic inflammation.

We also found that in the phylum Actinobacteria, the class Coriobacteriia and the family Coriobacteriaceae were positively correlated with body weight and proinflammatory cytokines, while the change in the proportions of these bacteria was significantly associated with age. Although the mechanistic effects of age on the Coriobacteriaceae are unknown, it is positively associated with both ROS and inflammatory cytokines, which contribute to metabolic dysfunction [28, 29]. Furthermore, our study showed that the proportion of the genus *Pseudoflavonifractor* (phylum Firmicutes) was influenced by diet and there was significant interaction between diet and age. Although little information on *Pseudoflavonifractor* is available, a previous study showed that bacteria in this genus express class IV alcohol dehydrogenase, which is involved in butyrate synthesis [30].

A previous study showed a progressive increase in the abundance of Firmicutes in both HFD-fed and *ob/ob* mice with aging [11]. In humans, the ratio of Firmicutes to Bacteroidetes changed across life stages, and a higher ratio of Firmicutes to Bacteroidetes was observed in adults [31]. These results suggest that host age is an important factor that can affect the composition of the gut microbiota. The reason for the respective higher and lower proportions of Firmicutes and Bacteroidetes in older animals is not well understood. However, evidence has suggested that age induces intestinal immunosenescence and these age-related declines in immune function are closely linked to the increased growth of pathogenic bacteria, leading to a state of chronic inflammation [32]. Immunosenescence and chronic inflammation might therefore be responsible for age-related changes in the gut microbiota [33]. Our study showed that the relative abundance of the phylum Firmicutes was influenced by age and positively correlated with proinflammatory cytokines, with an inverse relationship between Firmicutes and tight junction protein claudin-1. These data suggest that the increases of both Firmicutes and Actinobacteria are able to stimulate colonic macrophages in their expression of pro-inflammatory cytokines, such as TNF-α, IL-1β, and IL-6.

Additionally, the components of the phylum Bacteroidetes, including the family Bacteroidaceae and the genus *Bacteroides*, were also affected by age and negatively associated with colonic proinflammatory cytokines, representing a positive correlation between these bacteria and tight junction proteins. Studies have shown that the relative abundances of the Bacteroidaceae and Ruminococcaceae families were decreased with aging in humans [34], whereas in rabbit, the abundance of the Bacteroidaceae was decreased with age and the Ruminococcaceae became the dominant taxon [35]. These data indicate that Bacteroidaceae abundance is strongly associated with age, while Ruminococcaceae abundance might be influenced by other factors, such as species, gender, and dietary composition. In our study, the proportion of family Ruminococcaceae (class Clostridia) was influenced by both diet and age. A previous study showed that mice fed a HFD (60% fat) for 12 weeks exhibited a significantly lower proportion of Ruminococcaceae than mice fed a low-fat diet (13% fat) [9]. The Ruminococcaceae are known to produce butyrate, which is an important energy source for colon cells [36]. Samples of the fecal microbiota from NAFLD patients contain a lower proportion of Ruminococcaceae than healthy subjects [37]. Therefore, the observed HFD- and age-induced decreases in Ruminococcaceae, in association with lower butyrate production, may be a contributing factor in obesity- and age-related metabolic disorders.

In this study, age also significantly decreased the proportion of Rikenellaceae. Although less information is available for Rikenellaceae, a previous study reported that the relative abundance of Rikenellaceae was negatively associated with calprotectin levels [38]. As elevated calprotectin is associated with the migration of neutrophils to the gut mucosa [39], the decreased proportion of Rikenellaceae may be related to the increase in colonic inflammation. Further studies are needed to investigate the relationship between the Rikenellaceae family and colonic inflammation.

The limitations of this study are as follows. First, this study did not measure the absolute number of bacteria, but instead analyzed their relative proportions in the total bacterial population. Secondly, we used relatively small number of fecal samples per group in the consideration of cost-effectiveness.

## Conclusions

Taken together, our data suggests that host age and dietary fat intake are important elements that induce proportional changes in the gut microbiota, and that these changes are associated with systemic inflammation. This study provides evidence that both age and diet change the gut microbiota composition within a short period of time. However, the exact roles of specific gut microbes in the development of obesity are still unknown. Further studies are needed to investigate the cause-effect relationships between specific bacterial species and metabolic complications, to better understand the function of gut microbiota and to provide effective therapeutic strategies for obesity-related chronic diseases.

## Methods

### Animal care

Five-week-old male C57BL/6 J mice were purchased from Central Laboratory (Seoul, Korea). All animals were housed in plastic cages, with 4–5 mice per cage, under constant temperature (23 ± 2 °C), humidity (50 ± 10%), and a 12-h light/dark cycle. After a 1-week acclimatization period, the mice were randomly assigned to one of five groups (group 1, mice sacrificed at week 0 (*n* = 15); group 2, mice fed the ND (15% of calories from fat) for 10 weeks (*n* = 15); group 3, mice fed the HFD (45% of calories from fat) for 10 weeks (*n* = 15); group 4, mice fed the ND for 20 weeks (*n* = 15); and group 5, mice fed the HFD for 20 weeks (*n* = 16)). The composition of the experimental diets was based on a modified AIN-93G diet, as shown in Table 2. The fat sources in the diet were corn oil and lard. Fresh diets were prepared every 2–3 days and stored in airtight containers at 4 °C in the dark. Food intake was monitored twice a week, and body weight was measured once a week. All care, maintenance, and experimental protocols were approved by the Institutional Animal Care and Use Committee of Sookmyung Women’s University (SM-IAUC-2013-0917-032).

**Table 2:** Major components of the experimental diets

Ingredients	Normal diet (g/kg)	High fat diet (g/kg)
Cornstarch	404	266.5
Dextrin	134.2	88.5
Sucrose	101.6	67.1
Fiber	50	50
Casein	198	240.4
L-cystine	3	3.7
Corn oil	12.44	45.36
Lard	49.76	181.44
Mineral mix	34.6	42.1
Vitamin mix	9.9	12
Choline bitartrate	2.5	3.1
*tert*-Butylhydroquinone	0.014	0.017
Total	1000.014	1000.217
Carbohydrate, % of energy	65.71	36.69
Protein, % of energy	19.30	19.30
Fat, % of energy	14.99	45.00
Total energy, kcal/kg	3728.30	4527.50

(Modified from Reeves et al., 1993) [40]

### Fecal and tissue sample collection

Fresh fecal pellets were obtained from individual mice in a clean cage prior to sacrifice. Stool samples were frozen immediately in liquid nitrogen, and stored at − 80 °C until assay. Animals were sacrificed at week 0 (*n* = 15), week 10 (*n* = 15 from each group), and week 20 (*n* = 15–16 from each group). At necropsy, animals were anesthetized with an intraperitoneal injection of a 2:1 mixture of Zoletil (Virbac, Magny-en-Vexin, France) and Rompun (Bayer, Seoul, Republic of Korea). Colon and liver samples were rapidly removed, rinsed with cold saline, and weighed. The colonic mucosa was laid flat on a glass slide, scraped with a second glass slide, frozen immediately in liquid nitrogen, and stored at − 80 °C until analysis.

### Real-time quantitative polymerase chain reaction analysis

Total RNA was extracted from the scraped colon mucosa using TRIzol® reagent (Invitrogen, Carlsbad, CA, USA) according to the manufacturer’s instructions. Total RNA (1 μg) was reverse-transcribed using a cDNA Synthesis Kit (Genepole, Gwangmyeong, Korea) according to the manufacturer’s instructions. Real-time quantitative polymerase chain reaction (PCR) was performed on a 7500 Fast Real Time PCR system (Applied Biosystems, Foster City, CA, USA) using a QuantiMix SYBR Kit (Genepole). The cycling conditions were as follows: 15 min at 95 °C, followed by 40 cycles of 15 s at 94 °C and 30 s at 72 °C. Primers for TNF-α, IL-1β, IL-6, claudin-1, E-cadherin, occludin, ZO-1, and β-actin were synthesized by Bioneer (Daejeon, Korea), and their sequences are shown in Table 3. The relative fold change was determined by the 2^-ΔΔCt^ (relative quantification) method. Target gene expression levels were normalized to the expression of β-actin.

**Table 3:** RT-qPCR primer sequences (5′ to 3′)

Gene	Primer sequence (5′ → 3′)
TNF-α	Forward: CATCTTCTCAAAATTCGAGTGACAA
	Reverse: TGGGAGTAGACAAGGTACAACCC
IL-1β	Forward: TCACAGCAGCACATCAACAA
	Reverse: TGTCCTCATCCTGGAAGGTC
IL-6	Forward: ACTCACCTCTTCAGAACGAATTG
	Reverse: CCATCTTTGGAAGGTTCAGGTTG
Claudin-1	Forward: TCTACGAGGGACTGTGGATG
	Reverse: TCAGATTCAGCTAGGAGTCG
E-Cadherin	Forward: CCACCAAAGTCACGCTGAATAC
	Reverse: GGAGTTGGGAAATGTGAGCAA
Occludin	Forward: ACTGGGTCAGGGAATATCCA
	Reverse: TCAGCAGCAGCCATGTACTC
ZO-1	Forward: AGGCTACCTTTGTATTCTC
	Reverse: TAGGGCACAGTATTGTATC
β-Actin	Forward: CTCCTACCACACCCATTCTCATCC
	Reverse: GCAATGCCTGGGTACATGGTGG

### Western blot analysis

To obtain enough tissue protein samples to quantify caludin-1, E-cadherin, and occludin, samples from 2 to 3 mice with most similar body weight were pooled together and managed as one sample. Thirty micrograms of sample proteins were electrophoresed through 7.5% SDS-PAGE and transferred to polyvinylidene difluoride membranes (Amersham, Arlington Heights, IL, USA). The transferred membrane was blocked using 2% skim milk to inhibit non-specific proteins, and treated with primary antibodies against claudin-1 (Invitrogen), E-cadherin (Invitrogen), occludin (Invitrogen), and β-actin (Sigma-Aldrich). Anti-mouse immunoglobulin G conjugated with alkaline phosphatase was used as the secondary antibody. Each protein band was then confirmed and quantified using an enhanced chemiluminescence system (Amersham, Arlington Heights, IL, USA). The integrity of band was quantified by Image J software (Ver. 1.46; NIH, Bethesda, MD, USA).

### Pyrosequencing

Fecal DNA was extracted using a QIAamp DNA Stool Mini Kit (Qiagen, Valencia, CA, USA) according to the manufacturer’s instructions (*n* = 3–5 from each group). The extracted metagenomic DNA was amplified using primers targeting the V1 to V3 regions of the 16S rRNA gene. For bacterial amplification, the barcoded primers 9F (5′-CCTATCCCCTGTGTGCCTTGGCAGTC-TCAG-AC-AGAGTTTGATCMTGGCTCAG-3′) and 541R (5′-CCATCTCATCCCTGCGTGTCTCCGAC-TCAG-X-AC-ATTACCGCGGCTGCTGG-3′) were used, where the underlined sequence is the target region of the primer and “X” indicates the unique barcode for each subject. The amplification was carried out under the following conditions: an initial denaturation step at 94 °C for 5 min, followed by 30 cycles of denaturation at 94 °C for 30 s, primer annealing at 55 °C for 30 s, and extension at 72 °C for 5 min and 20 s. PCR products were resolved by electrophoresis on 2% agarose gels and visualized using a Gel Doc system (BioRad, Hercules, CA, USA). Amplified products were purified with a QIAquick PCR Purification Kit (Qiagen). Equal concentrations of purified products were pooled prior to removal of short fragments (non-target products) with an AMPure bead kit (Agencourt Bioscience, Beverly, MA, USA). Product size and quality were assessed on an Agilent Bioanalyzer 2100 (Palo Alto, CA, USA) using a DNA 7500 chip. Mixed amplicons were produced by emulsion PCR, and then deposited on Picotiterplates. Pyrosequencing was carried out by ChunLab, Inc. (Seoul, Korea) with a GS Junior Sequencing system (Roche, Branford, CT, USA).

### Pyrosequencing data analysis

Pre-processing and taxonomic assignment of sequencing reads were conducted as described previously [41–43]. First, the sequencing reads from different samples were separated by their unique barcodes. Then, the barcode, linker, and primer were removed from the original sequencing reads. Any reads containing two or more ambiguous nucleotides, having a low quality score (average score < 25), or a length shorter than 300 bp were discarded. Potential chimeric sequences were detected by the Bellerophon program, which compares the BLASTN search results between the forward half and reverse half sequences. After removing chimeric sequences, the taxonomic classification of each read was assigned using the EzTaxon-e database (https://www.ezbiocloud.net/) [43], which contains the 16S rRNA gene sequences of type strains with valid published names, as well as representative species-level phylotypes of both cultured and uncultured entries in GenBank with complete hierarchical taxonomic classification from phylum to species. To compare samples with different read sizes, random subsampling was conducted to equalize read size, and shared OTUs and weighted uniFrac distance matrix among the four sample groups were obtained with the XOR analysis tool and Fast UniFrac analysis function of the CL community software, respectively (ChunLab, Inc., Seoul, Korea).

### Statistical analysis

The statistical analysis was performed using SAS version 9.4 (SAS Institute Inc., Cary, NC, USA). The results are expressed as means ± standard error (SEM). Student’s t-test was used to determine the statistical differences between ND and HFD groups. Two-way ANOVA was used to determine the effects of diet and age, and the interaction between diet and age. For the analysis of similarities, one-way ANOSIM test based on the UniFrac distance was performed by using anosim function of mothur package with 10,000 permutations [44]. The Pearson’s correlation coefficient was used to analyze correlations between gut microbiota composition and colonic expressions of biomarkers or body weight. All *P* values were calculated using two-sided tests, and a *P* value less than 0.05 was considered statistically significant.

## Data Availability

All data generated or analyzed during this study are included in this manuscript. The datasets used and/or analyzed during the current study are available from the corresponding author upon reasonable request.

## Abbreviations

ANOSIM: Analysis of similarities
HFD: High-fat diet
ND: Normal diet
OTUs: Operating taxonomic units
PCoA: Principal coordinate analysis
